# White Matter Integrity Is Associated With Intraindividual Variability in Neuropsychological Test Performance in Healthy Older Adults

**DOI:** 10.3389/fnhum.2019.00352

**Published:** 2019-10-15

**Authors:** Drew W. R. Halliday, Jodie R. Gawryluk, Mauricio A. Garcia-Barrera, Stuart W. S. MacDonald

**Affiliations:** ^1^Department of Psychology, University of Victoria, Victoria, BC, Canada; ^2^Institute on Aging and Lifelong Health, University of Victoria, Victoria, BC, Canada

**Keywords:** diffusion tensor imaging (DTI), intraindividual variability (IIV), dispersion, Alzheimer’s Disease Neuroimaging Initiative (ADNI), cognitive aging, neuropsychological assessment

## Abstract

Inconsistency of performance across neuropsychological testing instruments (dispersion) shows sensitivity to acquired injury and neurodegenerative pathology in older adults. The underlying neural correlates have remained speculative however, in spite of known white matter degradation seen in conjunction with elevated inconsistency in related operationalizations of intraindividual variability. Consistently, these operationalizations have controlled for artifactual age-related variance to increase measurement sensitivity of CNS dysfunction. In this study, dispersion was examined alongside composite scores of memory and executive functioning from the Alzheimer’s Disease Neuroimaging Initiative (ADNI). Forty-four healthy older adults (*M* = 72.0, *SD* = 6.4) underwent Diffusion Tensor Imaging (DTI) and neuropsychological testing spanning a range of cognitive domains. The results replicated previous findings, demonstrating reduced microstructural integrity with advanced age and increased integrity in high memory and executive functioning performers, across all major white matter tracts. With age first regressed from the composite scores, significant associations remained between greater executive functioning scores and greater microstructural integrity in the genu of the corpus callosum, right anterior corona radiata, anterior, posterior and rentrolenticular parts of right internal capsule, as well as right posterior thalamic radiation. With age regressed from the dispersion scores, greater values were primarily associated with decreased white matter integrity in the body and genu of corpus callosum, anterior corona radiata bilaterally and left superior longitudinal fasciculus. Dispersion is easily computed across speeded and accuracy-based measures and shows promise in detecting white matter damage, beyond that seen in the typical aging process. This appears to be the first investigation of neural correlates associated with increased dispersion.

## Introduction

Intraindividual variability (IIV) in cognitive behavioral performance has emerged as a sensitive metric in the assessment of various developmental and neuropathological phenomena. IIV has historically been neglected in favor of using scores based on central tendency, in order to attenuate the impact of single scores (e.g., trials within a task, tests within a battery) that deviate from an individual’s mean performance. IIV is most typically operationalized in terms of response time inconsistency (RTI), with greater RTI having been linked to advanced age (Vasquez et al., 2016), impaired cognitive status (Murtha et al., 2002; Halliday et al., 2017), poorer cognitive function (Grand et al., 2016) and more rapid cognitive decline (Bielak et al., 2010) in older adults. Increased RTI has also been directly linked with reduced whole-brain white matter volume (Walhovd and Fjell, 2007) and to brain injury (Stuss et al., 2003). Accordingly, IIV has been posited as a sensitive metric to central nervous system integrity, including structural, functional and neuromodulatory correlates (e.g., MacDonald et al., 2006).

Dispersion is a related operationalization of IIV that reflects inconsistency in performance across a profile of multiple tasks (rather than within a single task). Greater dispersion reflects greater inconsistency across different tasks and has been understudied by comparison. Although such profiles are more likely to undergo scrupulous interpretation for etiology-specific presentations in clinical practice (e.g., for differential diagnosis of vascular relative to Alzheimer’s dementia), this level of analysis is less feasible in other contexts (e.g., brief neuropsychological screening). Instead, the initial identification of elevated dispersion that may be interpreted as irrelevant scatter (i.e., unusual patterns of responding), may instead assist clinicians in triaging clients for more comprehensive evaluation. Like RTI, dispersion has shown increases in late-life (Christensen et al., 1999) and has been associated with greater incidence of dementia in cross-sectional (Halliday et al., 2018) and population-based longitudinal studies of older adults (Holtzer et al., 2008). In addition to processes associated with normative aging and neurodegeneration, greater dispersion has also been linked to acquired neurological insults in postconcussive college athletes (Rabinowitz and Arnett, 2013), suggesting that it may be sensitive to a wide range of neurological insults. In spite of the demonstrated utility of dispersion thus far, the underlying neural correlates have not been directly examined.

Neuroimaging presents opportunities to understand potential neural correlates of cognitive function, without necessarily relying on patient populations and clinical phenomena to elucidate relationships of interest. Diffusion Tensor Imaging (DTI) is a magnetic resonance imaging (MRI) technique that measures the extracellular diffusion of water molecules and is used to infer properties of white matter microstructure. Fractional anisotropy (FA) is commonly used as an index of microstructural integrity of white matter, especially in white matter tracts (Feldman et al., 2010); higher values represent greater microstructural integrity. Mean diffusivity (MD) is sensitive to a microstructural loss due to cellularity, edema and necrosis (e.g., due to axonal loss; Alexander et al., 2011); higher values represent less overall integrity. Cognitive aging studies using whole-brain histograms have demonstrated an anterior-to-posterior and a superior-to-inferior gradient of decline in white matter integrity (Sullivan and Pfefferbaum, 2006; Davis et al., 2009; Madden et al., 2009; Zahr et al., 2009), in addition to increases in MD as a function of increasing age (Madden et al., 2009). This suggests that white matter integrity changes in a predictable fashion in later life; however, associations with cognitive performance have been challenging to interpret, in general. Indeed, calls for additional understanding of structural-functional and anatomical-clinical relationships of important white matter tracts (e.g., superior longitudinal fasciculus, uncinate fasciculus) have been raised to better understand their functional characterizations with regard to higher-order cognitive function (e.g., executive functioning—EF; Markis et al., 2004). Emerging evidence supports the notion that tracts connecting frontal areas (e.g., genu, uncinate fasciculus) are heavily associated with EF (Davis et al., 2009; Ziegler et al., 2010; Smith et al., 2011; Voineskos et al., 2012) and tracts connecting temporal areas are heavily associated with memory (Ziegler et al., 2010).

In a review of 18 studies examining associations between white matter integrity and cognitive performance in healthy older adults, Madden et al. (2009) found that composite scores for different cognitive domains demonstrated correlations of similar magnitude and across a similar range of white matter tracts, relative to individual tasks within a given cognitive domain. Although in theory, white matter should correlate strongly with measures of information processing speed (Nilsson et al., 2014; Kuznetsova et al., 2016), task impurity remains an important consideration (Madden et al., 2009). As a single and representative score for a given cognitive domain, composite scores have historically been advantageous in that they reduce the total number of degrees of freedom required for downstream analytic purposes, while also reducing measurement error and minimizing the contribution of any one particular score. In theory, composite indices attenuate the impact of scores that deviate from an individual’s mean performance across similar tasks, in service of a more robust, representative index. In doing so, however, they may also obfuscate variance that is otherwise meaningful (e.g., dispersion), given that the magnitude and consistency of deviate scores have shown sensitivity to CNS insults.

As a purported marker of neurological insult, the neural underpinnings of dispersion in cognitive performance have yet to be examined, in spite of mounting indirect evidence from clinical populations that dispersion is sensitive to neural integrity. The present investigation sought to examine the associations between white matter integrity with both composite and dispersion scores based on neuropsychological test performance in healthy older adults. *A priori* hypotheses included that both dispersion and composite scores would be sensitive to white matter integrity. It was further anticipated that memory scores (dispersion and composite) would be associated with memory-specific white matter tracts (e.g., fornix, temporal lobe white matter) and that EF scores would be more heavily associated with frontal-parietal white matter tracts (e.g., superior longitudinal fasciculus). Memory and EF domains were of particular interest, given the relevance of these domains in day-to-day functioning (Lezak, 1982; Jurado and Rosselli, 2007; Baggetta and Alexander, 2016), as well as in cognitive aging and cognitive decline (Grady, 2008; Vaughan and Giovanello, 2010). Given robust findings of age-related decline in white matter, we further anticipated that age would attenuate linkages between white matter integrity and neuropsychological performance.

## Materials and Methods

Data used in the preparation of this article were obtained from the Alzheimer’s Disease Neuroimaging Initiative (ADNI) database^1^. The ADNI was launched in 2003 as a public-private partnership, led by Principal Investigator Michael W. Weiner. The primary goal of ADNI has been to test whether serial MRI, positron emission tomography (PET), other biological markers, and clinical and neuropsychological assessment can be combined to measure the progression of mild cognitive impairment (MCI) and early Alzheimer’s disease (AD).

### Participants

A full description of the eligibility criteria for the ADNI project are outlined in the procedures manual (Alzheimer’s Disease Neuroimaging Initiative, 2012). The ADNI study enrolls participants between the ages of 55 and 90 from 57 sites across the United States and Canada. All participants that were included in the current study were from the cognitively normal (healthy control) group. Specifically, all participants in the current study did not report memory concerns and did not show any impairments in cognitive function or performance of activities of daily living. According to the ADNI protocol, participants in this group must have normal memory function, as evidenced by a Logical Memory II subscale score of ≥9 with 16+ years of education on the revised Wechsler Memory Scale, a Mini-Mental State Examination score from 24 to 30 and a Clinical Dementia Rating of 0. Individuals with subjective memory concerns, MCI or AD, as defined by the National Institute of Neurological and Communicative Disorders and Stroke and the AD Related Disorders Association diagnostic criteria, were excluded. The ADNI-2 protocol was updated from previous versions to include DTI at specific sites with 3T GE MRI scanners. Of the approximately 150 healthy control participants enrolled in ADNI-2, the current study retrieved all of the DTI data available for healthy controls at the screening time point, which yielded 44 participants/scans. Further information about inclusion and exclusion criteria used across the ADNI study can be found in the procedures manual^2^. The selected sample (*n* = 44) ranged between 59 and 89 years of age (*M* = 72.0, *SD* = 6.4), 93% of which were right-handed, and 56.8% of which were female. A more titrated age selection was not implemented in order to maximize degrees of freedom. Participant education ranged between 12 and 20 total years (*M* = 16.3, *SD* = 2.7). All ADNI participants provided informed written consent, which was approved by each site’s institutional review board.

### MRI and DTI Scanning

According to ADNI protocol, images were acquired from 3T MRI scanners (GE Medical Systems). Axial diffusion weighted image data were acquired with a spin echo planar imaging sequence. Scan parameters were as follows: acquisition matrix = 256 × 256, voxel size = 1.4 × 1.4 × 2.7 mm^3^, flip angle = 90°, number of slices = 59. There were 46 images acquired for each scan: 41 diffusion-weighted images (*b* = 1,000 s/mm^2^) and five non-diffusion-weighted images (*b* = 0 s/mm^2^). Repetition time varied across scanning sites but was approximately 13,000 ms.

### Image Analysis

#### Preprocessing Steps

Analyses were performed using Functional MRI of the Brain Software Library (FSL) version 5.0.10 (Analysis Group, FMRIB, Oxford, UK^3^; Jenkinson et al., 2012; Smith et al., 2004). In order to correct for head motion and eddy distortions, raw diffusion weighted imaging (DWI) volumes were aligned to the average b_0_ image using the *eddy_correct* tool (Andersson and Sotiropoulos, 2016). Non-brain tissue (e.g., skull, CSF) was subsequently removed using the *brain_extraction* tool, with corresponding images visually analyzed to inspect for accuracy.

#### Image Analysis

To obtain a projection of each participants’ FA data onto a mean skeleton, DTIFit was used to create FA maps, which were subsequently used for Tract-Based Spatial Statistics (TBSS; Smith et al., 2006). TBSS facilitates aligning FA images from multiple individuals onto an alignment-invariant representation for subsequent analyses, in order to improve the overall objectivity and interpretability of results. Individual FA data were non-linearly aligned to a common space (FMRIB58_FA) and a mean FA image was created and thresholded (FA > 0.2), in order to produce the mean FA skeleton. Individual FA and MD data were then projected onto the mean FA skeleton. The *randomise* tool was subsequently used to perform voxelwise statistical analyses on the white matter skeleton, using threshold-free enhancement to correct for multiple comparisons (*p* < 0.05, one-tailed). Within-person analyses were computed to examine associations with the cognitive scores of interest, with white matter regions identified through the John Hopkin’s University ICBM DTI-81 atlas (Mori et al., 2008).

### Dispersion Index

**Figure 1:**
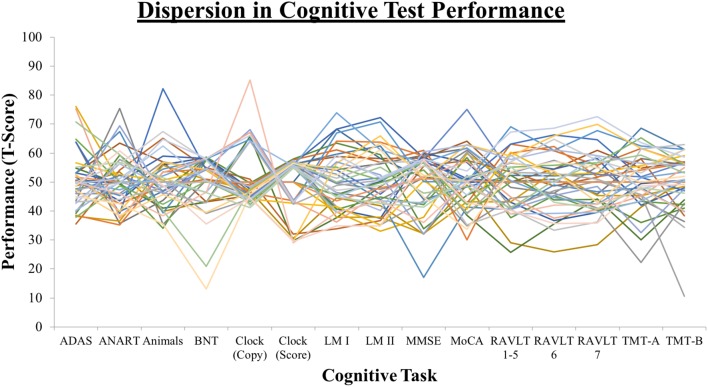
Dispersion profiles depicting the overall magnitude of variability across the cognitive testing profile.

Dispersion is a measure of intraindividual variability across domains of cognitive performance that is computed as an intraindividual standard deviation (ISD), reflecting performance fluctuations across a profile of test measures within an individual. Dispersion profiles were derived using a regression technique, which computes ISD scores from standardized test scores (Christensen et al., 1999; Hultsch et al., 2002). Test scores of interest were initially regressed on linear and quadratic age trends to control for age differences in mean performance, given that greater variance tends to be associated with greater means (Hale et al., 1998; Stawski et al., 2019) and that mean-level performance is likely to differ across the age range present in the current sample. Raw test scores were chosen and where necessary, were re-ordered so that high scores were uniformly indicative of better performance. We included tests that overlapped as much as possible with previously established composite scores described in the following section. These tests were selected from the time point corresponding most closely to the DTI measurements and are listed in Table 1, along with an indication of the lowest and highest recorded scores. The resulting residuals from these models were standardized as T-scores (*M* = 50, *SD* = 10), with ISDs subsequently computed across these residualized test scores. The resulting dispersion estimate, indexed on a common metric, reflects the amount of variability across an individual’s neuropsychological profile relative to the group average level of performance; higher values reflect greater IIV in cognitive function. Dispersion scores were computed across all test scores in the battery (broad profile dispersion: ISD-BP *M* = 9.12, *SD* = 2.57), as well as within memory (ISD-Mem *M* = 7.07, *SD* = 3.78) and EF (ISD-EF *M* = 8.80, *SD* = 7.75) domains. One case was excluded pairwise on the basis of having only five of the available fifteen test scores, resulting in a dispersion score greater than 3 SD above the group mean. Figure 1 depicts the overall magnitude of the broad profile dispersion score within the sample. Number of tests completed was not significantly associated with dispersion scores (*p*s > 0.05).

**Table 1:** List of neuropsychological tests included in the dispersion computations, as well as the lowest and highest scores observed.

Cognitive domain	Neuropsychological Test	Score	Lowest	Highest
Memory	Alzheimer’s Disease Assessment Scale-Cog (ADAS-Cog)	Total	1	20
	Logical Memory (WMS-R based on Story A)	LM I	8	20
		LM II	7	18
	Rey Auditory Verbal Learning Test (RAVLT)	A1–5	18	63
		A6	0	15
		A7	0	15
Executive function	Category fluency (animals and vegetables)	Total	13	37
	Clock Draw	Copy	3	5
		Score	3	5
	Trail Making Test A (TMT-A)	Total (seconds)	14	61
	Trail Making Test B (TMT-B)	Total (seconds)	36	222
Global cognition	Montreal Cognitive Assessment (MoCA)	Total	41	70
	Mini-Mental State Examination (MMSE)	Total	24	30
Language	American National Adult Reading Test (ANART)	Total	18	30
	Boston Naming Test (BNT)	Total	0	30

*LM, Logical Memory; WMS-R, Wechsler Memory Scale-Revised*.

### Composite Indices

The AD Neuroimaging Initiative Memory (ADNI-Mem, comprising the RAVLT, LM I, LM II, ADAS-Cog 3, and MMSE; Crane et al., 2012) and Executive Functioning (ADNI-EF, comprising Category fluency, Clock draw, Digit span backwards, Digit Symbol Substitutions, Number Cancellation, TMT-A and TMT-B; Gibbons et al., 2012) scores were established in previous investigations as composite measures for each respective domain, based on advanced psychometric analyses (e.g., item response theory). In validating the composite scores, each score was subsequently examined in terms of sensitivity to conversion to demented cognitive status, and in association to structural (e.g., white matter) and functional (e.g., CSF) biomarkers. Thus, the ADNI-Mem and ADNI-EF composite scores (referred to as COM-Mem and COM-EF hereafter) represent a suitable avenue to compare dispersion scores against in the present investigation. Notably, the tests comprising the dispersion and composite scores did not overlap entirely; however, we maximized this overlap as much as possible with what was available through the ADNI database. Number of tests completed was not significantly associated with COM-Mem or COM-EF (*p*s > 0.05).

## Results

### Correlations

Bivariate correlations were computed to examine the relationship between cognitive scores and chronological age (Table 2). Based on Cohen’s convention (Cohen, 1988), strong correlations were observed between the COM-Mem and COM-EF composite scores (*r* = 0.62, *p* < 0.001). Similarly, strong correlations were observed between ISD-BP with ISD-Mem (*r* = 0.49, *p* < 0.001) and ISD-EF (*r* = 0.58, *p* < 0.001), but not between ISD-Mem and ISD-EF (*r* = −0.10, *p* > 0.05). Significant correlations were also observed between age and COM-Mem (*r* = −0.58, *p* < 0.01), as well as COM-EF (*r* = −0.51, *p* < 0.01), such that older age was strongly associated with lower scores on each composite score. Age was not reliably associated with the dispersion scores (ISD-BP, ISD-Mem or ISD-EF), all *p* > 0.05. Moderate correlations were observed between COM-Mem and ISD-Mem (*r* = −0.30, *p* < 0.05, one-tailed), such that participants who performed more consistently on memory tasks also had higher memory composite scores. Without age regression performed prior to deriving the dispersion scores, the correlation patterns were quite similar. Without age regressed, ISD-BP, ISD-Mem and ISD-EF were not reliably associated with age (all *p*s > 0.05). Similarly, ISD-Mem and ISD-EF were not reliably associated with COM-Mem or COM-EF, when age was not first regressed.

**Table 2:** Bivariate correlations amongst cognitive scores and chronological age.

	Age	COM-Mem	COM-EF	ISD-BP	ISD-Mem
COM-Mem	−0.58**				
	*n* = 41				
COM-EF	−0.51**	0.62**			
	*n* = 41	*n* = 41			
ISD-BP	0.15	−0.15	−0.11		
	*n* = 41	*n* = 41	*n* = 41		
ISD-Mem	0.24	−0.30^+^	−0.19	0.49**	
	*n* = 40	*n* = 40	*n* = 41	*n* = 45	
ISD-EF	−0.13	−0.03	−0.03	0.58**	−0.10
	*n* = 45	*n* = 41	*n* = 40	*n* = 40	*n* = 40

Alzheimer’s Disease Neuroimaging Initiative (ADNI) memory (COM-Mem) and executive functioning (COM-EF) scores, without age regressed. Intraindividual standard deviation (ISD) for the broad profile (ISD BP) and within memory (ISD Mem) and executive functioning (ISD EF) domains, with age regressed. **p < 0.001; ^+^*p* < 0.05, one-tailed.

### White Matter Associations With Age

Age was examined in association with FA and MD, based on TBSS analyses. Negative associations were observed between FA and age across all major white matter tracts at the 95% threshold for significance (critical value = 0.994). In contrast, no positive associations were observed between FA and age (critical value = 0.010). Positive associations were observed between MD and age across all major white matter tracts at the 95% threshold for significance (critical value = 0.996). In contrast, no negative associations were observed between MD and age (critical value = 0.000). Taken together, these results replicate previous findings demonstrating reduced whole-brain microstructural white matter integrity with advanced age (e.g., Madden et al., 2009). Next, we examined the associations between the composite and dispersion scores with FA and MD; first without age regressed from the scores and then with age regressed.

### White Matter Associations With Composite Scores

The memory and EF composite scores (COM-Mem and COM-EF) were examined in association with FA and MD, based on TBSS analyses (see Table 3 for a summary of critical values). Positive associations were observed between FA and both the COM-Mem and COM-EF composite scores, across all major white matter tracts at the 95% threshold for significance, with no significant negative associations observed. Negative associations were observed between MD and both the COM-Mem and COM-EF composite scores, across all major white matter tracts at the 95% threshold for significance, with no significant positive associations observed. Taken together, these results replicate previous findings demonstrating that increased microstructural integrity is associated with better cognitive performance in both memory and executive functioning across all major white matter tracts (Crane et al., 2012; Gibbons et al., 2012; Nir et al., 2013).

**Table 3:** Critical values at which the threshold-free cluster enhancement (TFCE) statistic can be thresholded to yield a familywise error rate of 5% for fractional anisotropy (FA) and mean diffusivity (MD).

Variable	Association	Fractional Anisotropy		Mean Diffusivity	
		Uncorrected	Corrected	Uncorrected	Corrected
COM-EF	Positive	0.980*	0.942*	0.000	0.302
	Negative	0.000	0.050	0.984*	0.672
COM-Mem	Positive	0.925*	0.756	0.014	0.542
	Negative	0.114	0.126	0.994*	0.700
ISD-EF	Positive	0.416	0.694	0.110	0.064
	Negative	0.100	0.152	0.638	0.692
ISD-Mem	Positive	0.603	0.352	0.380	0.728
	Negative	0.495	0.600	0.180	0.082
ISD-BP	Positive	0.196	0.320	0.982*	0.946*
	Negative	0.823	0.320	0.038	0.232

Values are depicted for the analyses when the cognitive scores are both corrected and uncorrected for age-related variance. *p < 0.05, one-tailed.

### White Matter Associations With Composite Scores Controlling for Age

**Figure 2:**
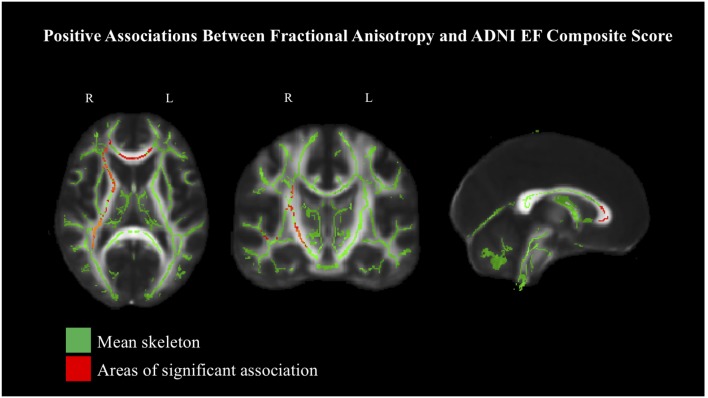
Positive associations between COM-EF and white matter, based on fractional anisotropy (FA) at 90% threshold, depicted in radiological view. Mean FA skeleton shown in green. Areas of significant association shown in red.

Given the strong associations between age and both white matter integrity and the ADNI composite scores, additional analyses were performed with age first regressed from COM-Mem and COM-EF, in order to better understand the associations between white matter integrity and cognitive performance. The previously significant associations between COM-Mem and COM-EF with FA and MD were attenuated well below significance in all instances, except for COM-EF and FA (see Figure 2 and Table 3 for a summary of critical values). Significant positive associations at the 90% threshold for significance between COM-EF and FA were observed primarily in the genu of the corpus callosum (11.8% of voxels), right anterior corona radiata (13.0% of voxels), anterior (15.2% of voxels), posterior (12.4% of voxels) and rentrolenticular parts of right internal capsule (33.8% of voxels), as well as right posterior thalamic radiation (6.3% of voxels).

### White Matter Associations With Dispersion

Each dispersion score (ISD-BP, ISD-Mem and ISD-EF) was examined in association with FA and MD, based on TBSS analyses (see Table 3 for a summary of critical values). No significant associations were observed between ISD-Mem or ISD-EF with FA or MD. Similarly, no significant associations were observed between ISD-BP and FA; however, positive associations were observed with MD in posterior (left: 22.2% of voxels; right: 24.5% of voxels), superior (left: 51.4% of voxels; right: 32.2% of voxels) and anterior corona radiata (left: 23.9% of voxels; right: 23.0% of voxels), bilaterally; anterior limb of the internal capsule, bilaterally (left: 74.0% of voxels; right: 79.9% of voxels); external capsule, bilaterally (left: 57.7% of voxels; right: 64.2% of voxels); bilateral fornix (left: 15.8% of voxels; right; 32.2% of voxels); genu of the corpus callosum (68.4% of voxels); and inferior longitudinal fasciculus, bilaterally (left: 38.9% of voxels; right: 46.7% of voxels). In these tracts, greater intraindividual variability across neuropsychological test performance corresponded with decreased microstructural integrity in these areas. To facilitate comparison with the following set of analyses, these results are reported at the 90% threshold for significance.

### White Matter Associations With Dispersion Controlling for Age

**Figure 3:**
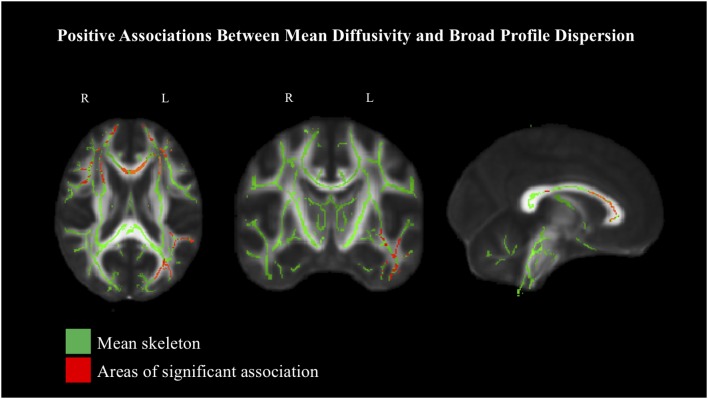
Positive associations between intraindividual standard deviation (ISD)-BP and white matter, based on mean diffusivity (MD) at 90% threshold, depicted in radiological view. Mean FA skeleton shown in green. Areas of significant association shown in red.

Each dispersion score (ISD-BP, ISD-Mem and ISD-EF) was examined in association with FA and MD, based on TBSS analyses (see Table 3 for a summary of critical values). Given *a priori* hypotheses regarding expected directional associations with dispersion, we employed one-tailed tests (*p* < 0.05) for specific planned comparisons. No significant associations were observed between the ISD-Mem or ISD-EF scores with FA or MD. Similarly, no significant associations were observed between ISD-BP and FA; however, positive associations were observed with MD primarily in the body (4.0% of voxels) and genu of the corpus callosum (17.7% of voxels), anterior corona radiata bilaterally (left: 20.6% of voxels; right: 17.2% of voxels) and left superior longitudinal fasciculus (7.8% of voxels) at the 90% threshold for significance (Figure 3), indicating that greater intraindividual variability across neuropsychological test performance corresponded with decreased microstructural in these areas. Few associations were reliable at the 95% threshold for significance.

## Discussion

As a measure of IIV, dispersion across cognitive test performance has shown promise as a sensitive index of CNS integrity, with emerging evidence from both acquired (e.g., concussion) and neurodegenerative impairments (e.g., dementia). In contrast to the more typically employed operationalization of IIV (RTI), dispersion has been comparatively understudied and the neural underpinnings have yet to be directly examined. The primary aims of the present study were to examine associations between white matter integrity using DTI with dispersion in performance across a broad battery of neuropsychological tests in older adults. We also sought to replicate previous findings examining white matter associations with age and cognitive performance based on composite scores.

The reported results replicate a number of these previous findings in older adulthood. Strong associations were observed between increased age with reduced microstructural integrity, across all major white matter tracts (Sullivan and Pfefferbaum, 2006; Davis et al., 2009; Madden et al., 2009; Zahr et al., 2009). Using the ADNI-Mem and ADNI-EF composite scores to index aggregate memory and EF performance, respectively, we also replicated previous findings demonstrating that greater microstructural integrity was associated with better performance in both cognitive domains (Crane et al., 2012; Gibbons et al., 2012). Dispersion within memory and EF areas was not reliably associated with white matter, contrary to anticipation; however, increased broad profile dispersion, reflecting larger amounts of intraindividual variability across participants’ neuropsychological test battery, was associated with lower white matter integrity. Interestingly, although most effects were observed bilaterally, greater dispersion was associated with reduced integrity in left longitudinal fasciculus, but not right. This may stem from processes that are typically left-hemisphere dominant and common across neuropsychological tests (e.g., language, right motor function for graphomotor output in right handed individuals) and therefore the relative importance of left longitudinal fasciculus for successful performance. That reliable white matter associations were only observed with dispersion across the entire battery (and not with the domain-specific dispersion indices) may result from the fact that meaningful inconsistency in cognitive performance requires a sufficient number of indicators that was otherwise not afforded in the more circumscribed indices.

Amongst the cognitive scores, results in general suggest that the composite and dispersion indices are associated with one another in general, but that there is little association *across* the two different operationalizations. The memory composite and dispersion scores were moderately correlated with one another, such that greater aggregate memory performance was associated with greater consistency of performance across memory tasks. The same pattern was not observed between the EF composite and dispersion scores. Composite scores correlated strongly with age, whereas dispersion scores did not, and this was the case even when age was not regressed from the dispersion scores initially. With age-regressed, the dispersion approach yields a score that is theoretically more purely associated with CNS function from the outset, given that age-related artifact (i.e., mean-level performance) is directly accounted for. In contrast, researchers employing the composite scores have typically accounted for age at later stages of analysis (Crane et al., 2012; Gibbons et al., 2012).

As is the case with composite scores, in utilizing multiple samples within and across cognitive domains, dispersion scores also afford greater reliability than any one test in isolation, while providing an overall snapshot of neurological status that confers information which may be orthogonal to that of composite scores. Unlike related operationalizations of IIV (e.g., RTI), dispersion can be computed based on both speed and accuracy-based measures with sufficient range, rendering the approach more ecologically feasible in applied settings. Importantly, dispersion does not capture where an individual’s mean level of performance across tests falls in relation to the group mean; that is, an individual may show the same magnitude of dispersion, with mean performance above or below the group mean. Thus, although dispersion may increase the overall sensitivity in identifying individuals with mild neurological impairment (e.g., early MCI, mild TBI), it is not suitable as an alternative to metrics that inform the directionality of performance (e.g., mean). Nevertheless, individuals who are *consistently* high- or low-performing across cognitive domains are likely closer to their own baseline state, relative to individuals who demonstrate elevated dispersion. Individuals with elevated dispersion may, therefore, be less capable of compensating in areas that are beginning to decline, relative to their otherwise typical levels of performance.

### Limitations and Future Directions

Replication of the reported findings will be essential to solidify the utility of composite and dispersion scores for indexing white matter integrity. Additional protocols available in future waves of the ADNI study (e.g., the brain health registry questionnaire^4^) may further contribute to a more in-depth understanding of these relationships. Although the sample was rigorously screened (e.g., for medical complications and cognitive impairment) and evenly represented the older adulthood age range, the effects reported here may nevertheless differ in the general population. The dispersion computation accounts for age-related artifact, where test scores are regressed on both linear and quadratic age trends and are then converted to T-scores; however, these scores are nevertheless derived within-sample. Future studies employing dispersion may consider employing appropriate population-based norms (e.g., Canadian Study of Health and Aging, Mayo’s Older Americans Normative Studies) to derive T-scores to examine the congruence of effects using both approaches (i.e., the more controlled within-sample approach relative to the more generalizable population-based approach). Future studies may also consider examining dispersion in different populations (e.g., Alzheimer’s Disease, Multiple Sclerosis), and within complex multidimensional domains such as executive functioning (e.g., Karr et al., 2018) to better understand the utility of the approach with respect to different neuropsychological phenomena.

## Conclusion

Dispersion is easily computed across speeded and accuracy-based measures and shows promise in detecting white matter damage, beyond that seen in the typical aging process. This investigation is the first to demonstrate that inconsistency across neuropsychological test performance is associated with white matter integrity in multiple brain regions. Although some clinicians may be inclined to interpret such inconsistency as merely scatter, a reliably quantified index of inconsistency helps remove this subjective bias, leaving open the possibility for detection of at-risk individuals who may benefit from continued monitoring. This may be especially important for individuals who do not present with marked deficits on any one particular task, but instead present with multiple, mild deficits that may otherwise go unnoticed.

## Data Availability Statement

The datasets generated for this study are available on request to the corresponding author.

## Ethics Statement

All ADNI participants provided informed written consent, which was approved by each site’s institutional review board.

## Author Contributions

DH and JG contributed to conceptualization, hypothesis development, data analysis and manuscript writing. MG-B and SM contributed to conceptualization, hypothesis development and manuscript writing.

## Conflict of Interest

The authors declare that the research was conducted in the absence of any commercial or financial relationships that could be construed as a potential conflict of interest.

## Footnotes

^1^adni.loni.usc.edu

^2^https://adni.loni.usc.edu/wp-content/uploads/2008/07/adni2-procedures-manual.pdf

^3^http://fsl.fmrib.ox.ac.uk

^4^https://www.brainhealthregistry.org/
